# Propranolol can induce PTSD‐like memory impairments in rats

**DOI:** 10.1002/brb3.905

**Published:** 2018-01-18

**Authors:** Rong‐Ting Zhu, Xiang‐Hui Liu, Yan‐Wei Shi, Xiao‐Guang Wang, Li Xue, Hu Zhao

**Affiliations:** ^1^ Faculty of Forensic Medicine Zhongshan School of Medicine Sun Yat‐Sen University Guangzhou China; ^2^ Guangdong Province Key Laboratory of Brain Function and Disease Zhongshan School of Medicine Sun Yat‐Sen University Guangzhou China; ^3^ Guangdong Province Translational Forensic Medicine Engineering Technology Research Center Zhongshan School of Medicine Sun Yat‐Sen University Guangzhou China

**Keywords:** β‐adrenoceptor, contextual fear conditioning, hippocampus, memory consolidation, post‐traumatic stress disorder

## Abstract

**Introduction:**

One hallmark symptom of post‐traumatic stress disorder (PTSD) is an inability to restrict fear responses to the appropriate predictor. An infusion of glucocorticoids (GCs) after a high‐intensity shock has been shown to induce PTSD‐like memory impairments. In addition to GCs, noradrenergic signalling is also recognized as a key biomarker underlying PTSD symptomatology.

**Methods:**

To explore the role of the noradrenergic system in PTSD‐like memory impairments, in this study, various doses of the β‐adrenoceptor antagonist propranolol were systemically or bilaterally injected into the dorsal hippocampus immediately after unpaired cue‐shock contextual fear conditioning, and then the rats were tested 24 h later.

**Results:**

Interestingly, we found that only low‐dose propranolol could induce PTSD‐like memory impairments, as rats showed reduced freezing to the correct predictor and generalized fear responses to the safe cues, accompanied by increased NE levels in the hippocampus and altered neural activity within the frontal‐subcortical circuit.

**Conclusion:**

These findings demonstrate that the noradrenergic system is involved in regulating the consolidation of contextual fear memory and that propranolol can dose‐dependently induce PTSD‐like memory impairments.

## 1 INTRODUCTION

Post‐traumatic stress disorder (PTSD) is associated with impaired processing of traumatic memories, increased psychiatric symptom severity, and functional disability (Geuze, Vermetten, de Kloet, Hijman, & Westenberg, 2009). A core symptom of PTSD is an excessive generalization of fear that is characterized not only by a strong response to a previously learned fearful cue but also by a debilitating failure to suppress fear responses even in the presence of cues that signal safety (Jovanovic, Kazama, Bachevalier, & Davis, 2012). Kaouane and colleagues (Kaouane et al., 2012) developed an animal behavioral model that evaluates the ability of subjects to restrict fear responses to the appropriate predictor of a threatening stimulus. They found that infusion of high‐dose glucocorticoids (GCs) after contextual fear conditioning with high‐intensity shock induced PTSD‐like memory impairments in animals, which were manifested as decreased freezing to the correct predictor and generalized fear responses to the cues that were normally not a relevant predictor of the threat. However, GCs have also been used to prevent PTSD; thus, the efficacy of GCs appears to depend on the dose and time of administration (Steckler & Risbrough, 2012). Actually, in addition to GCs, dysregulated norepinephrine (NE) signalling has also been identified as a key biomarker underlying PTSD symptomatology (Geracioti et al., 2001; Southwick et al., 1999). However, it remains unclear whether the central noradrenergic system is involved in the formation of PTSD‐like memory impairments.

Increased NE signalling was shown to significantly contribute to the activation of the HPA axis under stressful conditions. NE fibers primarily originate from the locus coeruleus (LC). The LC–noradrenergic system extends broad projections throughout the forebrain, providing dense innervation to different cerebral structures (Heidbreder & Groenewegen, 2003; Sara, 2009; Vertes, 2004), including the hippocampus, which is well recognized as crucial region involved in PTSD. In addition, the noradrenergic system in the hippocampus is involved in regulating the consolidation of contextual fear memory, and blockade of β‐adrenoceptors (β‐ARs) in the hippocampus impairs contextual fear memory (Ji, Wang, & Li, 2003). In particular, propranolol, a β‐AR antagonist, has also been used to prevent PTSD, but the results have not been consistently replicated.

In this study, the recently reported mouse model of glucocorticoid‐induced PTSD‐like memory impairments (Kaouane et al., 2012) was employed in rats. We hypothesized that the noradrenergic system in the hippocampus may be involved in regulating the consolidation of this novel contextual fear memory; thus, propranolol might impair the consolidation of the contextual fear memory. To our surprise, low‐dose propranolol not only failed to reduce the fear responses but also induced PTSD‐like memory impairments. The neural circuitry involved in PTSD‐like memory impairments is still not clear. Therefore, we continued to examine the possible brain regions that may exhibit neural circuitry activity change.

## 2 MATERIALS AND METHODS

### 2.1 Animals

For all experiments, male Sprague–Dawley rats (280–320 g) were purchased from the Experimental Animal Center at Sun Yat‐Sen University. They were singly housed in a light‐ (12/12 h light/dark cycle, lights on at 08:00 a.m.) and temperature‐controlled (23 ± 1°C) room with *ad libitum* access to water and food. All behavioral experiments were performed between 10:00 a.m. and 2:00 p.m. All experimental protocols were approved by the Animal Care and Use Committee of Sun Yat‐Sen University and were conducted in compliance with the US National Institutes of Health Guide for the Care and Use of Laboratory Animals.

### 2.2 Surgery

A minimum of 8 days before training, rats were stereotaxically implanted with bilateral guide cannulae. When each rat was fully anaesthetized with sodium pentobarbital (50 mg/kg of body weight, i.p.), its skull was secured in a stereotaxic frame (RWD, Shenzhen, China), and 22‐gauge stainless steel guide cannulae were implanted with the cannula tips located 1.5 mm above the dorsal hippocampus (dHPC) [coordinates: anteroposterior (AP), −3.4 mm from bregma; mediolateral (ML), ±1.7 mm from midline; dorsoventral (DV), −2.7 mm from skull surface], 3 mm above the medial prefrontal cortex (mPFC) [coordinates: AP, +3 mm from bregma; ML, ±0.5 mm from midline; DV, −2 mm from skull surface], or 2 mm above the basolateral amygdala (BLA) [coordinates: AP, −2.8 mm from bregma; ML, ±5.0 mm from midline; DV, −6.5 mm from skull surface], with the incisor bar located 3.3 mm below the interaural line, according to the atlas of Paxinos and Watson (Paxinos & Watson, 2005). The cannulae were secured with two anchoring screws affixed to the skull with dental cement. Steel stylets were inserted into the guide cannulas to maintain patency until the rats were subjected to infusions. Animals were acclimatized to the vivarium for at least 8 days prior to surgery. During this recovery period, the rats were handled three times for 1 min to habituate them to the infusion procedure and examine healing.

### 2.3 Behavioral apparatus and procedure

The animal model of PTSD‐like memory impairments was modified from a previously reported protocol (Kaouane et al., 2012).

#### 2.3.1 Apparatus

An automated rodent fear conditioning system (Coulbourn Instruments, Allentown, PA, USA) was used to record animal behavior. The top and two opposite sides of the box were composed of aluminum panels, and the other two sides were composed of transparent organic glass (rear wall and front door). The chamber was equipped with a floor composed of 18 steel rods connected to a precision‐regulated shocker (Coulbourn Instruments) through which foot shocks were delivered. The apparatus was enclosed in a ventilated and sound‐attenuated box. A software program (Graphic State, Coulbourn Instruments, Allentown, PA, USA) was used to control the sound and electrical shock settings and to collect, display, and store the experimental data for analysis. All stimuli were controlled by a computer software package. The training chamber was cleaned with 5% ethanol before and after each trial.

#### 2.3.2 Behavioral training

Briefly, two days before the beginning of fear conditioning, all rats were individually habituated to an opaque PVC chamber (30 cm × 24 cm × 21 cm) with an opaque PVC floor and a brightness of 100 lux daily for 4 min. The box was cleaned with 4% acetic acid before each trial (context a). This pre‐exposure allowed the rats to become familiar with the chamber used for the cue‐alone test. The animals were placed into a different context, a conditioning box (28 cm × 21 cm × 22 cm) at a brightness of 60 lux. The chamber was cleaned with 70% ethanol before each trial (context b).

**Figure 1:**
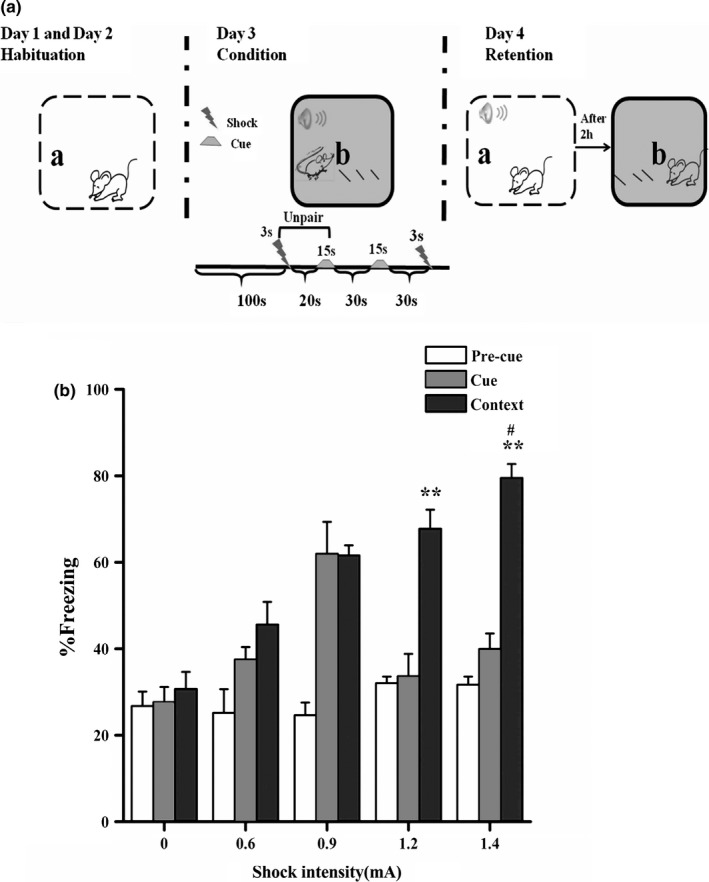
(a) Behavioral procedure. Day 1 and day 2, habituation in context ‘a’. Day 3, conditioning in context “b” with an unpaired tone and foot shock. Day 4, testing first in context “a” with the tone (1 kHz/2 kHz/white noise, WN) and then testing in context “b” without the tone. (b) An increasing shock intensity progressively produced higher fear responses to the correct predictor of the threat. ** *p *<* *.01 compared with 0.6 mA, ^**#**^ *p *<* *.05 compared with 1.2 mA (*n *=* *6–8 per group)

Contextual fear conditioning. Each animal was placed in “context b” for 4 min, during which it received two foot shocks (ranging from 0.6 to 1.4 mA for 3 s) (Atsak et al., 2012; Baldi, 2004) that never co‐occurred with two tones (1 kHz, 65 dB, 15 s). In this case, rats should identify the conditioning context and not the cue as the correct predictor of the shock (Kaouane et al., 2012) (Figure 1a).

Auditory fear conditioning. Each animal was placed in “context b” for 4 min. The tone cues (1 kHz, 65 dB, 15 s) was presented and immediately followed by two foot shocks (1.4 mA, 3 s) with an intertrial interval of 60 s. In this case, rats should identify the tone cue and not the conditioning context as the correct predictor of the shock. The procedures have been thoroughly described in a previous study (Kaouane et al., 2012).

#### 2.3.3 The open field tests

After twenty‐four hours, behavioral responses toward a novel context that was not associated with the contextual fear conditioning procedure were evaluated in open field arenas (dimensions: 50 × 50 × 50 cm). Animals were placed in the center. Measurements of locomotion—distance travelled and time spent in motion—were recorded using a digital video camera (Everio GZ‐MG275, Victor, Kanagawa, Japan) for 5 min, and locomotive behavior was analyzed offline using a software program (TopScan ver 2.00, Clever Sys., Inc., VA, USA). Open field arenas were wiped with 4% acetic acid between rats (Camp & Johnson, 2015; Ronzoni, Del, Mora, & Segovia, 2016).

#### 2.3.4 Memory retention tests

Twenty‐four hours after fear conditioning, rats were submitted to two memory retention tests. First, rats were placed in the safe and familiar chamber (context a) for 2 min for adaptation (precue test) and then exposed to the cue (65 dB, 1 kHz tone) for 2 min. Two hours later, rats were re‐exposed to the conditioning context (context b) for 2 min without the cue. Reversal of the order of the two tests produced equivalent results.

#### 2.3.5 Test for generalization

Twenty‐four hours after fear conditioning, separate groups of rats were placed in the safe and familiar chamber (context a) for 2 min for adaptation (pretone test) and then exposed to either the conditioning cue (65 dB, 1 kHz tone) or a novel tone (65 dB, 2 kHz tone) similar to the cue used during conditioning or to a very different novel tone (65 dB, white noise).

### 2.4 Drug administration

After conditioning, rats immediately received bilateral or systemic injections of drugs and were returned to their home cage. The nonselective β‐adrenoceptor antagonist DL‐propranolol hydrochloride (Sigma, St. Louis, MO, USA) was dissolved in saline vehicle (0.9%) and was injected subcutaneously or directly into the dHPC, BLA, or mPFC immediately after conditioning with 1.4‐mA foot shocks (Atsak et al., 2012). For systemic injections, the total volume of drug solution or equivalent volume of saline was 1 ml/kg. We examined the dose–response effects of propranolol (2 mg/kg, 5 mg/kg and 10 mg/kg). For bilateral infusions into specific brain regions, the injection needle was inserted into the guide cannula, with its tip located 1.5 mm beyond the end of the guide cannula. For bilateral infusions into the dHPC, propranolol was dissolved in saline (1.25 μg, 5 μg, 10 μg or 15 μg). Drugs were slowly infused through infusion cannulae at a rate of 0.5 μl/min using a CMA402‐automated syringe pump (CMA Microdialysis BA, Solna, Sweden). A total volume of 0.5 μl of the propranolol solution or an equivalent amount of saline vehicle was bilaterally infused into the dHPC. For bilateral infusions into the BLA and mPFC, the infusion procedure was similar to the infusions into the dHPC, with the exception of the volumes (0.2 μl for the BLA), doses (0.5 μg for the BLA, and 5 μg for the mPFC), and rates (0.2 μl/min for the BLA). The propranolol doses have been used in previous studies (Atsak et al., 2012; Cahill, Pham, & Setlow, 2000; Debiec & Ledoux, 2004; Quirarte, Roozendaal, & McGaugh, 1997; Reyes‐López, Nuñez‐Jaramillo, Morán‐Guel, & Miranda, 2010; Ronzoni et al., 2016; Villain et al., 2016). For bilateral infusions into the dHPC, the BLA or mPFC, the injection needles were retained within the cannulas for an additional 1 min after drug infusion to maximize diffusion and to prevent backflow of drug into the cannulas. All drug solutions were freshly prepared on the day of the experiment.

### 2.5 Immunohistochemistry

A separate cohort of rats underwent conditioning with the highest shock intensity (1.4 mA) and an intrahippocampal infusion of either saline or propranolol (5 μg per side). A group of naive rats, which were handled similarly and maintained in their home cages, were used as a control. Rats were then euthanized 90 min after the infusion to examine c‐Fos expression. Rats were deeply anaesthetized with sodium pentobarbital (50 mg/kg, i.p.) and perfused with formalin. Brains were quickly removed and postfixed overnight in the same fixative solution. Brains were embedded in paraffin and then sectioned in the coronal direction. Free‐floating sections were incubated in the primary polyclonal rabbit anti‐c‐Fos antibody (1/100; Abcam, Cambridge, UK) diluted in blocking solution for 36 hr at 4°C. Subsequently, sections were incubated with biotinylated goat anti‐rabbit IgG (1/2000; Jackson ImmunoResearch) for 2 hr at room temperature, followed by a 2‐hr incubation at room temperature with the avidin–biotin–peroxidase complex. The peroxidase reaction was performed by incubating sections with 3, 3′‐diaminobenzidine (DAB, Sigma, USA) for 2–10 min. Sections were mounted onto gelatine‐coated slides, dehydrated, cleared with xylene, and covered with neutral balsam. A video camera attached to a microscope and connected to analysis software was used to quantify the labelled cells. Sections from the CA1 and CA3 pyramidal layers and the DG granular cell layer were examined between −3.30 and −4.52 mm. Sections from the mPFC were quantified from bregma +3.60 mm to bregma +3.0. Sections from the BLA were quantified between bregma −2.8 mm and bregma −3.3 mm according to the atlas of Paxinos and Watson (Paxinos & Watson, 2005). For all brain regions, six measurements (three per hemisphere) were recorded from three different sections per rat and averaged. Measurements were also averaged between hemispheres for the mPFC and hippocampal subfields, but not for the amygdala, because we observed lateralization of c‐Fos expression. The computer automatically counted all positive targets in the regions of interest and excluded noncellular irregularities representing background staining. The experimenter was blinded to the experimental group. The results are expressed as the number of c‐Fos‐immunopositive cells per mm^2^ in each region.

### 2.6 Western blot analysis

Rats were deeply anaesthetized with sodium pentobarbital (50 mg/kg, i.p.). The mPFC, HPC subregions (CA1, CA3, and DG areas), and the BLA were dissected quickly from coronal slices, placed on ice under a dissecting microscope, and preserved in liquid nitrogen to avoid dephosphorylation and protein degradation. Tissues were homogenized in a freshly prepared lysis buffer supplemented with protease inhibitors and then centrifuged for 20 min at 12,000 rpm. The supernatant was then assayed for the total protein concentration using the BCA Protein Assay Kit. Samples were separated on 10% SDS–PAGE gels, and equal amounts of protein were fractionated by SDS–PAGE before electrotransfer to polyvinylidene difluoride (PVDF) membranes. Subsequently, Western blots were performed by blocking the membranes with 5% skim milk at room temperature for 1 h, cutting and then incubating the membranes with a specific rabbit anti‐c‐Fos antibody (1/1000; Abcam, Cambridge, UK) or rabbit anti‐GAPDH antibody (1/1000; Cell Signalling Technology, Danvers, MA, USA) overnight at 4°C. Membranes were washed with phosphate‐buffered saline containing 0.1% Tween (PBST), incubated with an HRP‐conjugated anti‐IgG antibody (1/2000; Applied Bio Probes, Rockville, MD, USA) at room temperature for 1 hr, and washed again, and im‐antibodies against soluble antigen‐reactive polypeptides were detected by chemiluminescence using ECL reagents (Millipore, Billerica, MA, USA) and subsequent autoradiography. Results were quantified by performing a densitometric scan of the films. Data were analyzed using ImageJ software by measuring the integrated density of the bands after subtracting the background.

### 2.7 Measurements of norepinephrine and epinephrine levels

Animals in which the dorsal hippocampus was injected with propranolol (5 μg per side) were sacrificed either under basal conditions or 30, 60, and 120 min after conditioning with 1.4‐mA shocks. Trunk blood and the dorsal hippocampus were quickly collected from animals subjected to each of the conditions. Rat hippocampal samples were dissected on dry ice and homogenized on ice to generate lysates (1 mg wet weight tissue into 40 μl of 0.01 N HCl, 1 mmol/L EDTA, and 4 mmol/L sodium metabisulfite). After centrifugation of blood in EDTA‐coated tubes at 2000 rpm for 10 min, the supernatant was stored at −80°C until the assay was performed. Norepinephrine was extracted from brain tissues according to the manufacturer's instructions, and the dried extracts were stored at −80°C until the assay was performed. Plasma and hippocampal levels of norepinephrine or epinephrine were determined by specific enzyme‐linked immunosorbent assays (ELISAs; Abnova, Taipei, Taiwan), according to the manufacturer's instructions.

### 2.8 Histology

After behavioral testing, rats were anaesthetized with an overdose of sodium pentobarbital and transcardially perfused with physiological saline, followed by 10% buffered formalin. Brains were removed from the skulls, postfixed with 4% paraformaldehyde at a low temperature for 1 week, and then placed in a 30% glucose solution. Brains were coronally sectioned at a thickness of 20 μm. Brain sections were mounted on gelatine‐coated glass slides and stained with thionin to evaluate the cannula placements.

### 2.9 Data analysis

Statistical analyses were performed using Student's t test or analysis of variance (ANOVA) followed by the Bonferroni's post hoc test when appropriate. Analyses were conducted using SPSS 20.0 software. *p* values <0.05 were considered statistically significant. All data in the text and figures are presented as the means ± SEM.

## 3 RESULTS

Fear conditioning to contextual cues was assessed by measuring freezing, a well‐established measure of conditioned fear in rats. In our experiments, we used a precue/tone test to reflect background freezing to the adaption context (as a result of possible generalization with the conditioning context) and found that none of the animals showed increasing fear responses to the adaption context in the precue/tone test. In memory retention tests, we did not observed differences when the order of the two tests was reversed such that rats were first re‐exposed to the cue or conditioning context.

### 3.1 An increasing shock intensity progressively produced increasing fear responses to the correct predictor of the threat

We first investigated the optimal shock intensity for the unpaired cue–shock contextual fear conditioning that allowed the rats to identify the conditioning context and not the cue as the correct predictor of the threat. During training, animals received foot shocks of different intensities (ranging from 0 to 1.4 mA); the rats exhibited greater fear responses to the correct predictor when they were re‐exposed to the context with the higher shock intensities (*F*_3, 22_ = 7.325, *p *=* *.001, one‐way ANOVA), and not to the cue. Specifically, Bonferroni's post hoc analysis indicated that the 1.2‐mA and 1.4‐mA shock groups showed conditioned fear responses to the correct predictor when re‐exposed to the context alone (1.2 mA, *p *=* *.003 and 1.4 mA, *p *<* *.001 compared to 0.6‐mA), but not to the cue (1.2 mA, *p *=* *1.000 and 1.4 mA, *p *=* *1.000 compared to 0.6‐mA). Furthermore, according to Student's t test, animals trained with the 1.4‐mA shock intensity produced greater fear responses to the context than animals trained with the 1.2‐mA shock (*F *=* *0.053, *t* (10)  = −2.435, *p *=* *.035) (Figure 1b). Thus, our findings indicate that 1.4 mA should be selected as the optimal shock intensity in the subsequent experiments.

### 3.2 An intrahippocampal infusion of propranolol dose‐dependently induced PTSD‐like memory impairments

**Figure 2:**
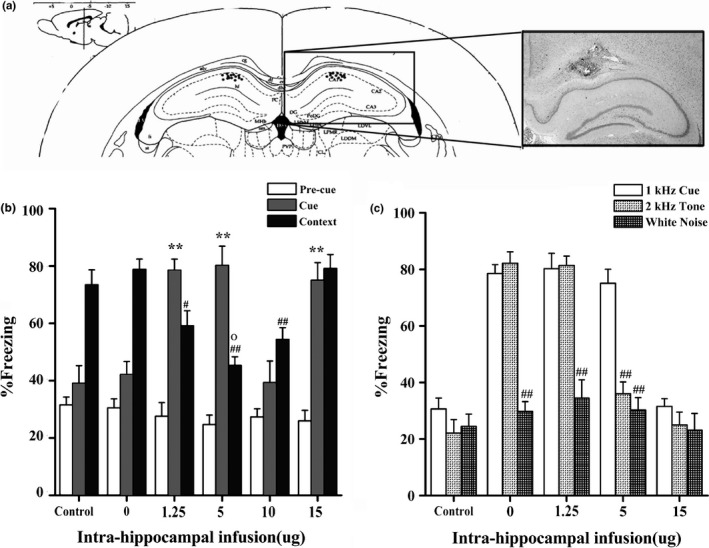
An intrahippocampal infusion of a low dose of propranolol‐induced PTSD‐like memory impairments. (a) Representative microphotograph depicting the injection site in the dHPC. (b) After a high‐intensity threat (1.4 mA), low doses of propranolol (1.25 or 5 μg) induced PTSD‐like memory impairments. ** *p *<* *.01, ^**##**^ *p *<* *.01 compared with the vehicle; ° *p *<* *.05, 5 μg versus 1.25 μg (*n *=* *6 per group). (c) An intrahippocampal infusion of propranolol (1.25 or 5 μg) increased the response to the 2‐kHz tone, but not to white noise. ^**##**^ *p *<* *.01 compared with the 1‐kHz cue (*n *=* *6 per group)

Previous reports indicate that the hippocampus is required for the formation and retrieval of contextual fear memories (Hall, Thomas, & Everitt, 2001), and β‐ARs in the hippocampus are involved in regulating the consolidation of contextual fear memories (Ji et al., 2003). In our experiments, the PTSD‐like memory impairment model is essentially recognized as a new kind of contextual fear conditioning. Hence, to investigate whether the noradrenergic system of the hippocampus is involved in regulating the consolidation of this contextual fear memory, vehicle or different doses of propranolol (1.25, 5, 10, or 15 μg in 0.5 μl per side) was bilaterally administered into the dorsal hippocampus immediately after conditioning with the high‐intensity threat (1.4 mA). To our surprise, as is shown in Figure 2b, a one‐way ANOVA indicated that propranolol induced dose‐dependent PTSD‐like memory impairments during retention testing, suppressing the response to the correct predictor (the context) (*F*_4,25_ = 14.850, *p *<* *.001) and increasing the fear response to the wrong predictor (the cue) (*F*_4,25_ = 16.285, *p *<* *.001). Based on Bonferroni's post hoc analysis, all doses of propranolol increased freezing responses to the cue (1.25 μg, *p *=* *.001; 5 μg, *p *<* *.001 and 15 μg, *p *=* *.002 compared with the vehicle group), except for the 10‐μg dose (*p *=* *1.000). In contrast, for the context, the lower dose groups (1.25, 5, and 10 μg), but not the highest dose group (15 μg, *p *=* *1.000 compared with that of the vehicle group), showed a significant reduction in freezing responses (1.25 μg, *p *=* *.01; 5 μg, *p *<* *.001; and 10 μg, *p *=* *.001 compared with the vehicle group).

In this study, the mouse model of PTSD‐like memory impairments (Kaouane et al., 2012) was adapted to rats. However, a previous study suggested that rats were not sensitive to low‐frequency sounds (Grothe & Pecka, 2014). Thus, the auditory fear conditioning was performed to check whether normal rats discriminate auditory tones of 1 and 2 kHz (Kaouane et al., 2012). Twenty‐four hours after auditory fear conditioning, normal rats can identify the cue (*F *=* *0.001, *t* (9) * *=* *−12.744, *p *<* *.001) and not the conditioning context (*F *=* *2.365, *t* (9) * *=* *−1.458, *p *=* *.179, individual t tests) as the correct predictor of the shock. In the generalization test, these rats exhibited an increased response to the 1‐kHz cue but not to the 2‐kHz tone (*F *=* *0.620, *t* (10) * *=* *9.831, *p *<* *.001) and white noise (*F *=* *2.499, *t* (10) * *=* *9.594, *p *<* *.001). Thus, the normal rats could discriminate auditory tones of 1 and 2 kHz (Figure S1).

Furthermore, after contextual fear conditioning, the control groups showed no fear response to a previously unexperienced 2‐kHz tone in the generalization test (the control group, *F *=* *1.062, *t* (8) * *=* *1.243, *p *=* *.249 compared with the 1‐kHz cue and the saline group, *F *=* *0.209, *t* (8) * *=* *1.388, *p *=* *.202 compared with the 1‐kHz cue), similar to the 1‐kHz tone experienced during conditioning and a completely different cue (white noise) (the control group, *F *=* *1.474, *t* (8) * *=* *1.297, *p *=* *.231 compared with the 1‐kHz cue and the saline group, *F *=* *0.010, *t* (8) =1.060, *p *=* *.320 compared with the 1‐kHz cue, individual t tests) (Figure 2c). Separate groups of rats that showed increasing fear responses to the 1‐kHz tone were included in the generalization test. As shown in Figure 2c, a one‐way ANOVA revealed differences in the animals’ fear responses to different cues (1.25 μg, *F*_2,15_ **=* *67.495, *p *<* *.001; 5 μg, *F*_2,15_ **=* *33.386, *p *<* *.001; 15 μg, *F*_2,15_ **=* *28.991, *p *<* *.001). Bonferroni's post hoc analysis revealed that animals treated with the 1.25‐μg and 5‐μg doses of propranolol showed fear responses to the unexperienced 2‐kHz tone (1.25 μg, *p *=* *1.00 and 5 μg, *p *=* *1.00 compared to the 1‐kHz cue) but not to the white noise (1.25 μg, *p *<* *.001 and 5 μg, *p *<* *.001 compared to the 1‐kHz cue). However, the fear response in 15‐μg dose group was exclusively restricted to the conditioning cue (2 kHz cue, *p *<* *.001 and white noise, *p *<* *.001 compared with the 1 kHz cue). In conclusion, low doses of propranolol (1.25 or 5 μg per side injected into the dorsal hippocampus) induce PTSD‐like memory impairments; the high dose (15 μg in 0.5 μl per side) of propranolol impairs the ability of the subject to restrict fear responses to the appropriate predicting cues but does not induce generalization. Meanwhile, only the moderate dose (10 μg in 0.5 μl per side) of propranolol effectively impairs the contextual fear memory.

In the open field tests, no significant effects of the different injection doses on overall locomotion—either on distance travelled (*F*_5,24_ **=* *0.831, *p *=* *.540, Bonferroni) or on time spent in motion (*F*_5,24_ **=* *0.559, *p *= .730, Bonferroni)—were observed (Figure S2). Thus, these findings indicate that the hippocampal noradrenergic system is involved in regulating the consolidation of contextual fear memory; intrahippocampal propranolol infusion can dose‐dependently induce PTSD‐like memory impairments without affecting general locomotion in the open field.

### 3.3 PTSD‐like memory impairments were associated with an altered neural activity within the mPFC–hippocampal–amygdalar circuitry

**Figure 3:**
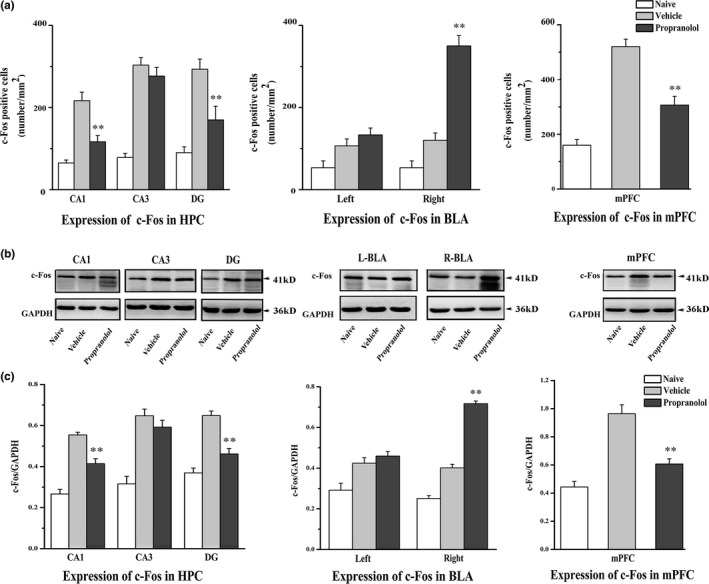
Injections of DL‐propranolol (5 μg) into the dorsal hippocampus altered neural activity within the frontal‐subcortical circuitry. (a) Immunohistochemical staining for the c‐Fos protein was performed ** *p *<* *.01, propranolol versus vehicle (*n *=* *6 per group) (Representative microphotographs of c‐Fos immunoreactivity in different groups are presented in Figure S3). (b and c) Western blot analyses of the expression of the c‐Fos protein were performed. ** *p *<* *.01, propranolol versus vehicle (*n *=* *5–6 per group)

Several studies have shown that the altered neural activity within the mPFC–hippocampal–amygdalar circuitry was involved in the pathophysiology of PTSD (Bailey, Cordell, Sobin, & Neumeister, 2013; Hendriksen, Olivier, & Oosting, 2014; Pitman et al., 2012). To measure the neurobiological mechanisms by which propranolol induced the PTSD‐like memory impairments, the expression of the c‐Fos protein was analyzed 90 min after an intrahippocampal infusion of either propranolol (5 μg per side) or vehicle following conditioning. A one‐way ANOVA showed significant differences in the number of c‐Fos‐expressing neurons in the examined brain regions between groups. The propranolol group, which identified the cue as a predictor of the shock, exhibited a significant decrease in c‐Fos expression in the dorsal CA1 (*F*_2,15_ **=* *28.475, *p *<* *.001; *p *=* *.001 compared with vehicle, Bonferroni after one‐way ANOVA), the DG (*F*_2,15_ **=* *20.211, *p *<* *0001; *p *=* *.009 compared with vehicle, Bonferroni), and the mPFC (*F*_2,15_ **=* *44.597, *p *<* *.001; *p *<* *.001 compared with vehicle, Bonferroni). Contextual fear conditioning is associated with lateralized expression of c‐Fos in the BLA (Scicli, Petrovich, Swanson, & Thompson, 2004), and an imbalance between the activity of the left and the right amygdala has also been reported to be involved in the development of PTSD (Smith, Abou‐Khalil, & Zald, 2008). For these reasons, levels of the c‐Fos protein expression were analyzed separately in the left and right amygdala. Consistent with previous findings, c‐Fos expression was increased in the right BLA in the propranolol group (*F*_2,15_ **=* *71.953, *p *<* *.001; *p *<* *0.001 compared with the vehicle, Bonferroni after one‐way ANOVA), but not the left BLA (*F*_2,15_ **=* *11.695, *p *=* *.001; *p *=* *.599 compared with the vehicle, Bonferroni). Moreover, no significant differences were observed in the CA3 region (*F*_2,15_ **=* *52.412, *p *<* *.001; propranolol versus vehicle, *p *=* *.853, Bonferroni) of the hippocampus between the propranolol and vehicle groups (Figure 3a).

In addition, we also used Western blot analyses to verify the results described above (Figure 3b,c). Consistent with the above‐mentioned findings, an intrahippocampal infusion of propranolol decreased c‐Fos expression in the dorsal CA1 (*F*_2,12_ **=* *35.402, *p *<* *.001; *p *=* *.004 compared with the vehicle, Bonferroni after one‐way ANOVA), the DG (*F*_2,13_ **=* *35.999, *p *<* *.001; *p *<* *.001 compared with the vehicle, Bonferroni), and the mPFC (*F*_2,13_ **=* *28.151, *p *<* *.001; *p *=* *.002 compared with the vehicle, Bonferroni) whereas increased c‐Fos expression in the right BLA (*F*_2,15_ **=* *157.442, *p *<* *.001; propranolol versus vehicle, *p *<* *.001, Bonferroni). No significant differences were observed in the CA3 (*F*_2,12_ **=* *18.679, *p *<* *.001; *p *=* *1.000 compared with the vehicle, Bonferroni) or the left BLA (*F*_2,15_ **=* *10.027, *p *=* *.002; *p *=* *1.000 compared with the vehicle, Bonferroni) between the two groups. Taken together, the DL‐propranolol (5 μg per side) injections into the dorsal hippocampus altered neural activity within the frontal‐subcortical circuitry.

### 3.4 DL‐propranolol administration increased NE levels in the dHPC, but not the plasma

**Figure 4:**
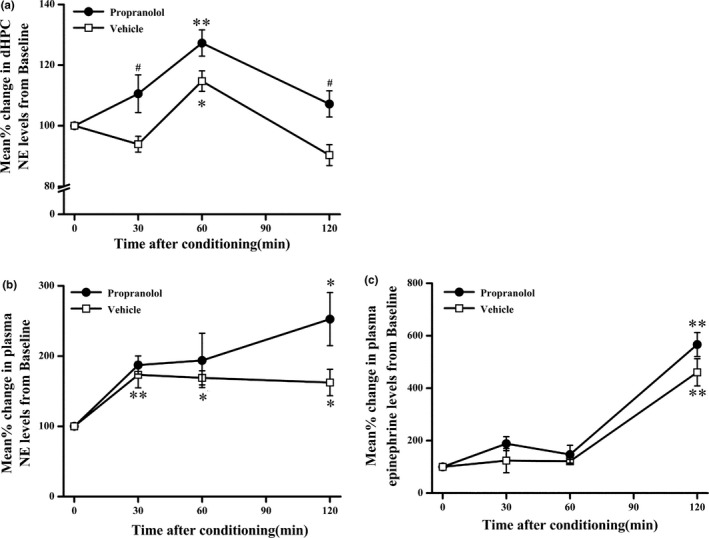
DL‐propranolol administration increased the NE levels in the dHPC, but not in the plasma. (a) Intrahippocampal injections of propranolol (5 μg) increased the NE levels in the dHPC. *p <* *.05, **p <* *.01 compared with the baseline; ^#^ *p *<* *.05 compared with the vehicle (*n *=* *4 per group). (b) Intrahippocampal injections of propranolol (5 μg) induced a similar increase in both NE and epinephrine levels in the plasma compared with the saline group. *p <* *.05, **p <* *.01 compared with the baseline (*n *=* *4 per group)

As described previously, dysregulated signalling mediated by the stress‐related neurotransmitter NE and a hypernoradrenergic state has been implicated in the pathophysiology of PTSD (Geracioti et al., 2001; Southwick et al., 1999; Strawn & Geracioti, 2008). A previous study suggested an inverted U‐shape relationship between the stress‐induced increase in the NE level in the hippocampus and contextual fear memory (Kao, Stalla, Stalla, Wotjak, & Anderzhanova, 2015). Thus, in this experiment, we investigated the levels of NE in the dHPC after the low‐dose intrahippocampal DL‐propranolol (5 μg) injection that induced PTSD‐like memory impairments. The NE concentrations in the four baseline samples collected prior to the control injections were averaged to yield the initial baseline value of 100%. Repeated‐measures ANOVA for NE levels at four consecutive time points showed significant effects of propranolol on treatment (*F*_1,6_ **=* *24.55, *p *=* *.003) and time (*F*_3,18_ **=* *15.429, *p *<* *.001), but not the interaction between propranolol treatment and time (*F*_3,18_ **=* *2.234, *p *=* *.119). Thus, NE levels changed throughout the course of the measurements collected after either saline or propranolol injection, and the NE levels in the propranolol group were higher than in the vehicle group. A one‐way ANOVA evaluated the fluctuations in hippocampal NE levels relative to baseline values across the different treatment periods (*F*_3,12_ **=* *15.354, *p *<* *.001 for vehicle; *F*_3,12_ **=* *7.009, *p *=* *.006 for propranolol). In the propranolol group, Bonferroni's post hoc test indicated that the NE concentrations in the 60‐min samples were significantly higher than the baseline levels (*p *=* *.005) after the propranolol injection; however, the NE concentrations in the 30‐min (*p *=* *.672) and 120‐min (*p *=* *1.000) samples were not significantly different from baseline values. Furthermore, in the saline group, the percent change in the NE levels collected after 60 min (*p *=* *.015, Bonferroni) was significantly greater than the baseline values, whereas those collected at 30 min (*p *=* *.859, Bonferroni) and 120 min (*p *=* *.169, Bonferroni) were not significantly different from baseline values. Individual t tests were used to assess differences in the NE concentrations between the vehicle and propranolol groups at each collection point throughout the experiment. The percent increase in the NE concentration observed in the propranolol group at 30 min (*F *=* *0.837, *t* (4) * *=* *2.473, *p *=* *.048) and 120 min (*F *=* *0.731, *t* (4) * *=* *3.053, *p *=* *.022) was significantly greater than the concentration in the saline group, but the difference at the 60‐min time point was not significant (*F *=* *0.423, *t* (4) * *=* *2. 282, *p *=* *.065) (Figure 4a).

Next, we investigated whether the intrahippocampal DL‐propranolol infusion (5 μg) affected plasma NE and epinephrine levels (Figure 4b,c). The concentrations of NE or epinephrine in the four baseline samples collected prior to the control injections were averaged to yield the initial baseline value of 100%. Repeated‐measures ANOVA at four consecutive (propranolol and vehicle groups) time points showed a significant effect of time (*F*_3,18_ **=* *9.868, *p *<* *.001 for NE; *F*_3,18_ **=* *154.202, *p *<* *.001 for epinephrine) but not of the interaction between propranolol treatment and time (*F*_3,18_ **=* *1.817, *p *=* *.180 for NE; *F*_3,18_ = 2.152, *p *=* *.129 for epinephrine) and propranolol treatment (*F*_1,6_ **=* *4.125, *p *=* *.089 for NE; *F*_1,6_ **=* *5.586, *p *=* *.056 for epinephrine), suggesting that plasma NE and epinephrine levels were not significantly different between the propranolol and vehicle groups. Thus, low‐dose intrahippocampal propranolol injections increased the NE levels in the hippocampus but not the plasma NE or epinephrine levels.

### 3.5 Functional involvement of specific brain areas in contextual fear memory

**Figure 5:**
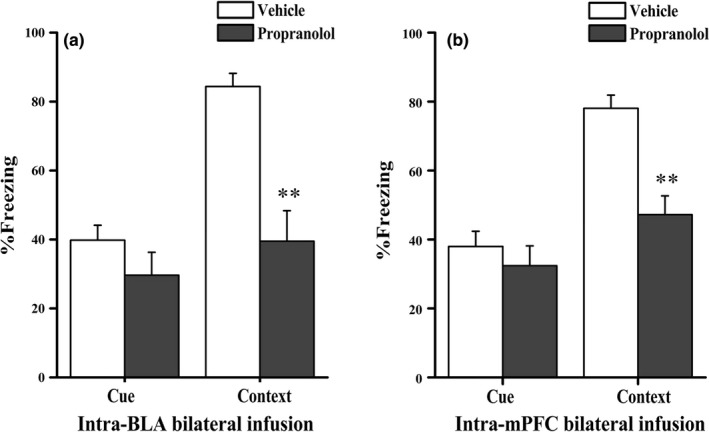
Effect of DL‐propranolol infusions into the BLA and mPFC on contextual fear memory consolidation. (a) Propranolol injections into the BLA impaired contextual fear memory consolidation (*n *=* *5 per group). (b) Propranolol injections into the mPFC impaired contextual fear memory consolidation. **p <* *.01 compared with the vehicle (*n *=* *5–7 per group)

In addition to the hippocampus, contextual memory also requires the participation of the amygdala and mPFC (Morilak et al., 2005; Stevenson, 2011). Moreover, our results regarding c‐Fos protein levels also indicated the involvement of the mPFC and amygdala in the PTSD‐like memory impairments. To investigate whether blockade of β‐ARs in the BLA or mPFC can also induce PTSD‐like memory impairments, different groups of animals were assigned to receive control or DL‐propranolol treatments directly into the BLA or mPFC after conditioning. As shown in Figure 5, individual t tests indicated that the intra‐BLA propranolol infusion significantly decreased freezing responses during the contextual retention test (*F *=* *5.083, *t* (8) * *=* *4.658, *p *=* *.002) but not during the cue retention test (*F *=* *3.317, *t* (8) * *=* *1.283, *p *=* *.235). In addition, the intra‐mPFC propranolol injection also decreased freezing responses during the contextual retention test (*F *=* *1.940, *t* (12) * *=* *4.711, *p *=* *.001), but not during the cue retention test (*F *=* *0.015, *t* (8) * *=* *0.772, *p *=* *.462). Accordingly, these data indicated that β‐adrenergic receptors in the mPFC and BLA are involved in regulating the consolidation of this contextual fear memory; however, blockade of β‐ARs in the BLA or mPFC did not induce PTSD‐like memory impairments (Figure 5a,b).

### 3.6 Low‐dose systemic DL‐propranolol administration induced PTSD‐like memory impairments

**Figure 6:**
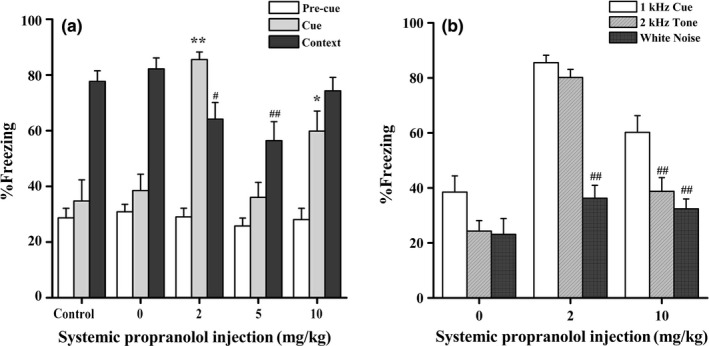
Effect of systemic DL‐propranolol injections on contextual fear memory consolidation. (a) After the high‐intensity threat (1.4 mA), systemic injections of a low dose of propranolol (2 mg/kg) induced PTSD‐like memory impairments. *p <* *.05, **p <* *.01, ^#^ *p *<* *.05, ^##^ *p *<* *.01, compared with 0 mg/kg (*n *=* *6–7 per group). (b) Effect of systemic propranolol (2 and 10 mg/kg) treatments on freezing behaviors during the retrieval of memories of different cue tests. *p <* *.05, **p <* *.01 compared with the 1‐kHz cue (*n *=* *5–7 per group)

Under physiological conditions, systemic norepinephrine levels increase in response to stress and play a crucial role in memory consolidation (Barsegyan, McGaugh, & Roozendaal, 2014; Soeter & Kindt, 2011). To better inform the development of targeted treatments and investigate the roles of the noradrenergic system in the formation of PTSD‐like memory impairments, rats received a systemic injection of either vehicle or different doses of propranolol (2, 5, or 10 mg/kg) immediately after the high‐threat (1.4 mA) contextual conditioning task. As shown in the results of the one‐way ANOVA presented in Figure 6a, the propranolol treatment induced a dose‐dependent change in the overall percent freezing behaviors during the retention test (*F*_3,19_ **=* *17. 863, *p *<* *.001 for cue retention; *F*_3,20_ **=* *6.423, *p *=* *.003 for contextual retention). Bonferroni's post hoc analysis revealed that freezing responses of both the 2‐mg/kg and 10‐mg/kg groups (*p *<* *.001 for 2 mg/kg, *p *=* *.026 for 10 mg/kg compared with vehicle), but not the 5‐mg/kg group (*p *=* *1.000), were significantly elevated in the cue retention test. However, in the contextual retention test, doses of 2 mg/kg (*p *=* *.049) and 5 mg/kg (*p *=* *.004) but not the higher dose of 10 mg/kg (*p *=* *1.000), significantly decreased freezing responses.

In addition, in the generalization test, the control groups did not show a fear response to a previously unexperienced 2‐kHz cue or a completely different cue (white noise). As shown in Figure 6b, animals treated with 2 mg/kg propranolol showed a similar fear response to the 2‐kHz tone (*F*_2,12_ **=* *57.645, *p *<* *.001; *p *=* *.930 compared to the 1‐kHz cue, Bonferroni after one‐way ANOVA), but did not show a response to the white noise (*p *<* *0.001). Animals treated with 10 mg/kg propranolol did not show any fear response to the 2‐kHz tone (*F*_2,17_ **=* *7.823, *p *=* *.004; *p *=* *.025 compared to the 1‐kHz cue, Bonferroni after one‐way ANOVA) or the white noise (*p *=* *.005). Accordingly, the systemic administration of the moderate dose of propranolol (5 mg/kg) effectively impaired this contextual fear memory consolidation, and the lowest dose (2 mg/kg) of propranolol induced PTSD‐like memory impairments in which the rats showed decreased freezing behaviors to the correct predictor and generalized fear responses to the safe cues. In summary, systemic DL‐propranolol administration impaired the consolidation of contextual fear memories and induced dose‐dependent PTSD‐like memory impairments.

## 4 DISCUSSION

This study provides important complementary evidence for the involvement of the noradrenergic system in regulating PTSD‐like memory impairments. In the present study, we used the unpaired cue–shock contextual fear conditioning animal model to show that low‐dose propranolol administered systemically or directly into the dorsal hippocampus after contextual fear memory conditioning not only failed to effectively reduce fear memory expression but also induced PTSD‐like memory impairments in which the rats exhibited decreased freezing responses to the correct predictor and generalized fear responses to the safe cues. Actually, the β‐AR antagonist propranolol has received considerable attention for its therapeutic potential in subjects with PTSD in previous studies, but contradictory pharmacological effects on both animal models and human participants have been reported. Propranolol has been shown to impair memory consolidation in some studies (Conversi, Cruciani, Accoto, & Cabib, 2014; Lonergan, Olivera‐Figueroa, Pitman, & Brunet, 2013) but not in others (Okamura et al., 2011; Palotai, Telegdy, Ekwerike, & Jaszberenyi, 2014). Thus, in view of these mixed results, it is becoming increasingly important to clarify the effects of propranolol in order to effectively guide treatment the development of treatments for PTSD.

Research in basic science and functional neuroimaging has helped to identify three brain regions that may be involved in the pathophysiology of PTSD: the amygdala, medial prefrontal cortex, and hippocampus (Shin, Rauch, & Pitman, 2006). A previous study suggested that the increased noradrenergic activity in the amygdala contributed to the expression of hyperarousal behavior in an animal model of PTSD (Ronzoni et al., 2016). As the hippocampus also receives noradrenergic afferents from the LC, low‐dose propranolol‐mediated blockade of β‐adrenoceptors in the hippocampus may alter neuronal activity and neurotransmitter release. Thus, we examined the changes in c‐Fos expression in these brain areas. In our study, the intrahippocampal infusion of a low dose of propranolol induced PTSD‐like memory impairments associated with decreased c‐Fos expression in the dorsal CA1, DG and the mPFC, and increased c‐Fos expression in the right BLA. To our knowledge, the hippocampus is not a uniform structure and consists of several subfields with specialized functions and distinctive histological characteristics, including the subiculum complex, the cornu ammonis sectors (CA1‐CA3), and the dentate gyrus (DG) (Lavenex & Banta, 2013). The CA1 is preferentially involved in contextual memory consolidation, whereas the CA3 region elaborates a unified representation of a context after the information is processed in the DG (Daumas, Halley, Frances, & Lassalle, 2005). The DG plays an important role in the discrimination of a similar context from a fearful one (Wu & Hen, 2014). Inhibition of dorsal DG increases the generalization of fear to an unfamiliar context that was similar to a feared context and impairs fear expression in the conditioned context when it was similar to a neutral context (Bernier et al., 2017). However, all subregions of the hippocampus are highly interconnected. Microinjections of a low dose of propranolol in the dorsal hippocampus induce different changes in c‐Fos expressions in hippocampal subregions via their internal neural pathways. In addition, based on the results from animal studies, stress‐related hippocampal damage primarily occurs in certain subfields (Duan, Kang, Liu, Ming, & Song, 2008; Shiryaeva, Vshivtseva, Mal’ Tsev, Sukhorukov, & Vaido, 2008). Specifically, we think the early use of low‐dose propranolol may selectively affect certain subfields, such as the CA1 and the DG, while sparing the CA3 region. These findings are consistent with previous evidence from patients with PTSD showing that pharmacological challenge tests or exposure to traumatic reminders are associated with increased noradrenergic responsiveness and hypoactive responses in the mPFC, as well as a hyperactive amygdala (Liberzon & Sripada, 2008; Shin et al., 2006). One neurocircuitry model of PTSD also posits that the amygdala is hyperresponsive, the medial prefrontal cortex is hyporesponsive, and the medial prefrontal cortex and the hippocampus fail to inhibit the amygdala (Layton & Krikorian, 2002).

During the acquisition of conditioned fear memories, the selection of the best predictor of the shock (tone versus context) requires different levels of activation of the hippocampal–amygdalar circuit (Trifilieff, Calandeau, Herry, Mons, & Micheau, 2007). It has been proposed that the hippocampus is used to assemble a contextual representation (e.g., odor, texture, illumination, and size of the environment) before that presentation is projected to the amygdala (Rudy, 2009). A key structure required for both context‐ and tone‐dependent fear conditioning (LeDoux, 2000) is the lateral nucleus of the amygdala, a critical site involved in the assessment of threat‐related stimuli, and/or biologically relevant ambiguity (Davis & Whalen, 2001; Morris, Ohman, & Dolan, 1998). According to our experiments, rats showed a fear response to a tone that was part of the stressful experience but was never associated with shock delivery and even a tone resembling the previous cue that had never been experienced. Similarly, excessive generalization of fear from a failure to discriminate dangerous from safe stimuli, a core symptom of PTSE has been proposed to be a biomarker of PTSD (Jovanovic et al., 2012). To investigate how neurons encode the switch from specific to generalized fear, a recent study (Ghosh & Chattarji, 2015) identified the cellular substrate in the amygdala required for the alteration of emotional states from normal to pathological fear. They observed that the same amygdalar neurons that displayed cue‐specific responses before the behavioral shift to generalized fear lost their specificity afterward, thereby tilting the balance of activity toward a greater proportion of generalizing neurons. The combined effect of the increase in danger‐evoked firing along with a larger increase in cue‐evoked spiking combined to enhance overall neuronal excitability in the amygdala, thereby explaining clinical reports on exaggerated amygdalar responses in patients with anxiety disorders. Furthermore, targeted activation of cAMP–PKA signalling in the amygdala increases the neuronal excitability of amygdalar neurons and produces generalized fear (Ghosh & Chattarji, 2015). These findings provide new insights into a major behavioral problem reported in patients with PTSD.

Interestingly, we also found that low‐dose propranolol injections into the dorsal hippocampus caused altered neural activity within the frontal‐subcortical circuitry, similar to the alterations observed in GC‐induced PTSD‐like memory impairments. Extensive evidence indicates that GCs interact with arousal‐induced noradrenergic activity to impair the retrieval of hippocampus‐dependent memory (Roozendaal, de Quervain, Schelling, & McGaugh, 2004; Roozendaal, Hahn, Nathan, de Quervain, & McGaugh, 2004). Moreover, previous studies suggested that propranolol administration elevated cortisol levels (Tollenaar, Elzinga, Spinhoven, & Everaerd, 2009; Viru et al., 2007). Viru and colleagues (Viru et al., 2007) proposed a possible mechanism by which the sympathetic nervous system plays a dual inhibitory and excitatory role in adrenocortical function: The inhibitory effects are designed to avoid exaggerated hormonal responses or an adjustment that occurs only in response to β‐blockade through which enhanced adrenaline production compensates for the reduced influence of NE that increases corticotropin‐releasing hormone (CRH) and adrenocorticotropic hormone (ACTH) levels, which in turn stimulates cortisol release. In another study, lower doses of propranolol were not sufficient to effectively block consolidation of memories of intensively stressful task involving a higher intensity of noradrenergic transmission because negative reinforcement was present and thus required higher doses of the β‐receptor antagonist than other tasks (Debiec & Ledoux, 2004).

PTSD is characterized by heightened noradrenergic signalling. High levels of NE release during exposure to a traumatic event have been proposed to result in overconsolidation of the traumatic memory, thereby leading to PTSD (Pitman, 1989). The increased NE transmission in the hippocampus may be involved in the pathophysiology of PTSD (Acheson, Gresack, & Risbrough, 2012). In the present study, we also observed elevated NE levels in the hippocampus of the group showing PTSD‐like memory impairments but the plasm NE and epinephrine levels were not altered. These results are consistent with evidence that NE levels in both the hippocampus and the mPFC are significantly higher in a PTSD group than in controls (Kao et al., 2015; Wilson, McLaughlin, Ebenezer, Nair, & Francis, 2014), and individuals who do and do not develop PTSD do not exhibit differences in in‐hospital plasma, salivary, or urinary cortisol or NE levels (Shalev et al., 2008; Videlock et al., 2008). However, researchers have not clearly determined why a local infusion of a low dose of propranolol elevated NE levels in the dHPC. The hippocampus is known to control the responses of the HPA axis via distinct pathways that connect the CA1 and subiculum with the hypothalamic nuclei and mammillary bodies (Tannenholz, Jimenez, & Kheirbek, 2014). A low‐dose intrahippocampal infusion of propranolol may have partially stimulated corticosterone release through a negative feedback mechanism, as described above (Viru et al., 2007), subsequently indirectly contributing to increased NE levels.

It has been well established that blockade of β‐ARs in different areas of the brain impairs memory consolidation (Ji et al., 2003; Morilak et al., 2005). To verify whether propranolol‐induced PTSD‐like memory impairments have brain region specificity, different groups of animals were assigned to receive DL‐propranolol treatments directly into the BLA or mPFC after training. We found that blockade of β‐ARs in the BLA or mPFC did not induce PTSD‐like memory impairments. Thus, the hippocampus is believed to be the main brain region responsible for the PTSD‐like memory impairments. In addition, we also found that propranolol dose‐dependently impaired the consolidation of contextual fear memories, as the intrahippocampal injection of a moderate dose (10 μg in 0.5 μl per side) of propranolol effectively impaired contextual fear memory, likely because the moderate dosage is sufficient to effectively block the consolidation of a memory of an intensively stressful task. The direct actions of propranolol elicited by localized infusions likely result in greater blockade of β‐receptor signalling that impedes memory consolidation. However, the highest intrahippocampal propranolol dose elevated the freezing response to an irrelevant cue and had no effect on contextual fear memory. The higher concentration of the drug may diffuse into the dental gyrus, which is highly sensitive to stress (Hunsaker & Kesner, 2008), producing a negative effect on memory consolidation through its actions in this region. The conflicting results might be due to differences in the doses of the drug or routes of administration (systemic or direct injections into different brain regions). Moreover, it would be interesting to examine histological and biochemical parameters in animals injected with a high dose (10 μg or 15 μg) of propranolol, which may reveal certain mechanisms that are responsible for the difference between the effects of low‐ and high‐dose injections.

In order to develop precision therapy, the effects of the systemic administration of propranolol on memory must be better characterized. The noradrenergic system is required for modulating memory processes, and stimulation of β‐ARs facilitates emotional memory consolidation (Barsegyan et al., 2014; Soeter & Kindt, 2011). A previous study has demonstrated that knockout mice lacking NE displayed impaired contextual fear memory; however, the memory was rescued by activation of hippocampal β‐ARs, but not α‐ARs (Murchison et al., 2004). Thus, β‐AR signalling is critical for contextual memory retrieval in NE knockout mice. In the present study, rats that received a systemic injection of propranolol exhibited dose‐dependent PTSD‐like memory impairments. It is generally known that DL‐propranolol is a highly lipophilic nonselective β‐adrenergic receptor blocker. The apparent discrepancy in the effects of the different doses of propranolol is likely due to differences in the concentrations of propranolol that cross the blood–brain–barrier and reach local brain regions, such as the hippocampus, BLA, and mPFC. It is reasonable to consider the potential treatment implications of the present findings.

## 5 CONCLUSIONS

To our knowledge, our results are the first to show that microinjections of a low dose of propranolol in the dorsal hippocampus can induce PTSD‐like memory impairments in animal. However, this effect of the propranolol injection was specific for contextual fear memory, and propranolol may have an overall effect on memory consolidation in other behavioral tasks. The nature and extent of the memory impairments remain to be determined to specifically and effectively target memories involved in the pathophysiology of disorders. In the future, further studies need to be performed to investigate the neurobiological mechanisms underlying the dose‐effect relationships of propranolol on the formation of PTSD‐like memory impairments.

## FINANCIAL DISCLOSURES

The authors have no competing financial interests to declare.
