# Supplementary material for: Propranolol can induce PTSD‐like memory impairments in rats
Source: Brain Behav. 2018 Jan 18;8(2):e00905. doi: 10.1002/brb3.905 (PMC5822589; doi:10.1002/brb3.905)
Supplement: Supplementary file 1 [file BRB3-8-e00905-s001.pdf]

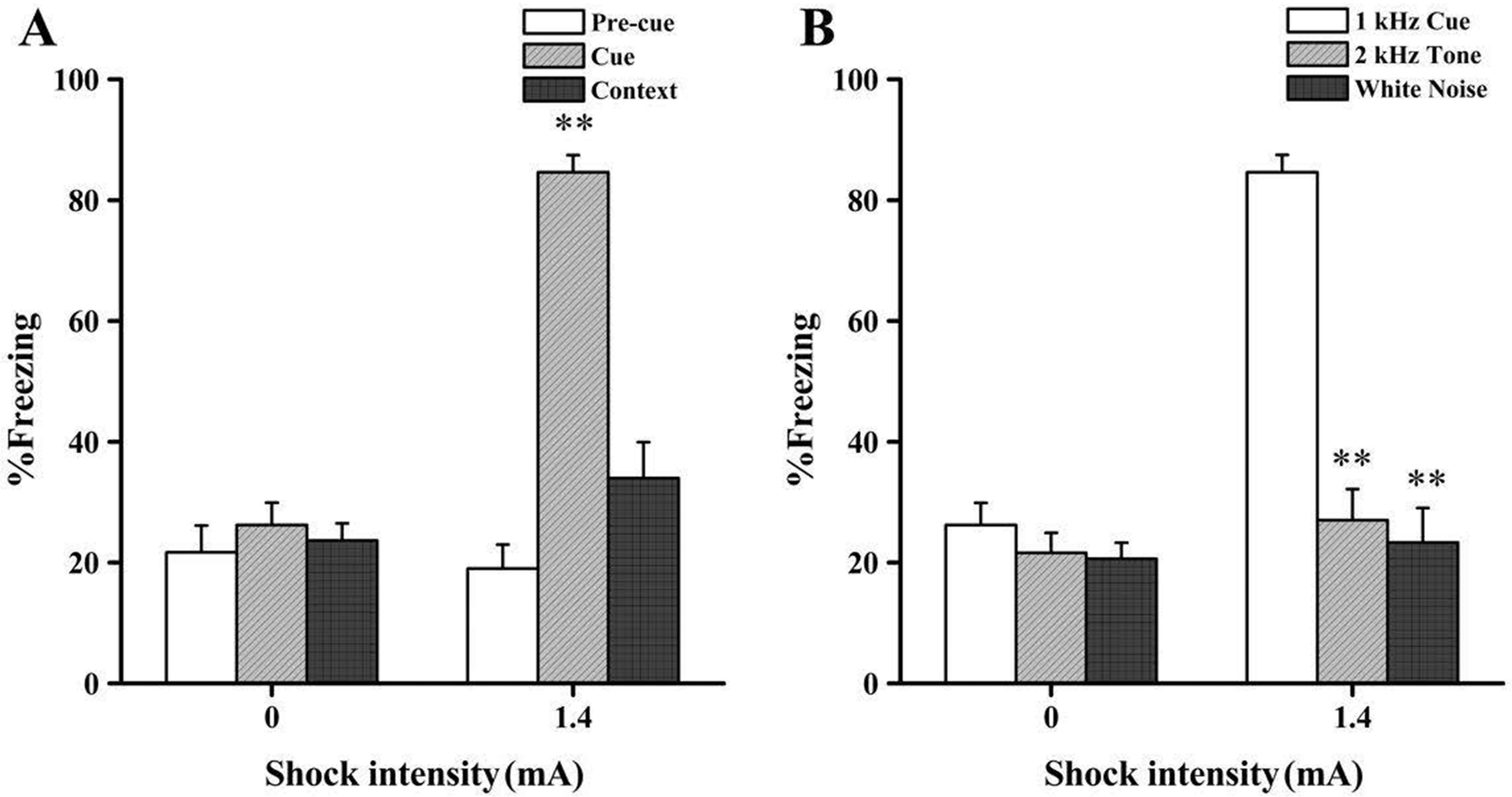

Fig. S1. Auditory fear conditioning. (A) Normal rats identified the cue and not the conditioning context as the correct predictor of the shock. ***P* < 0.01 compared with 0 mA. (B) Normal rats discriminated the auditory tones of 1 and 2 kHz. Rats exhibited an increased response to the 1-kHz cue, but not to the 2-kHz tone or white noise. ***P* < 0.01 compared to the 1-kHz cue (n = 5-6 per group).
