# Supplementary material for: Propranolol can induce PTSD‐like memory impairments in rats
Source: Brain Behav. 2018 Jan 18;8(2):e00905. doi: 10.1002/brb3.905 (PMC5822589; doi:10.1002/brb3.905)
Supplement: Supplementary file 2 [file BRB3-8-e00905-s002.pdf]

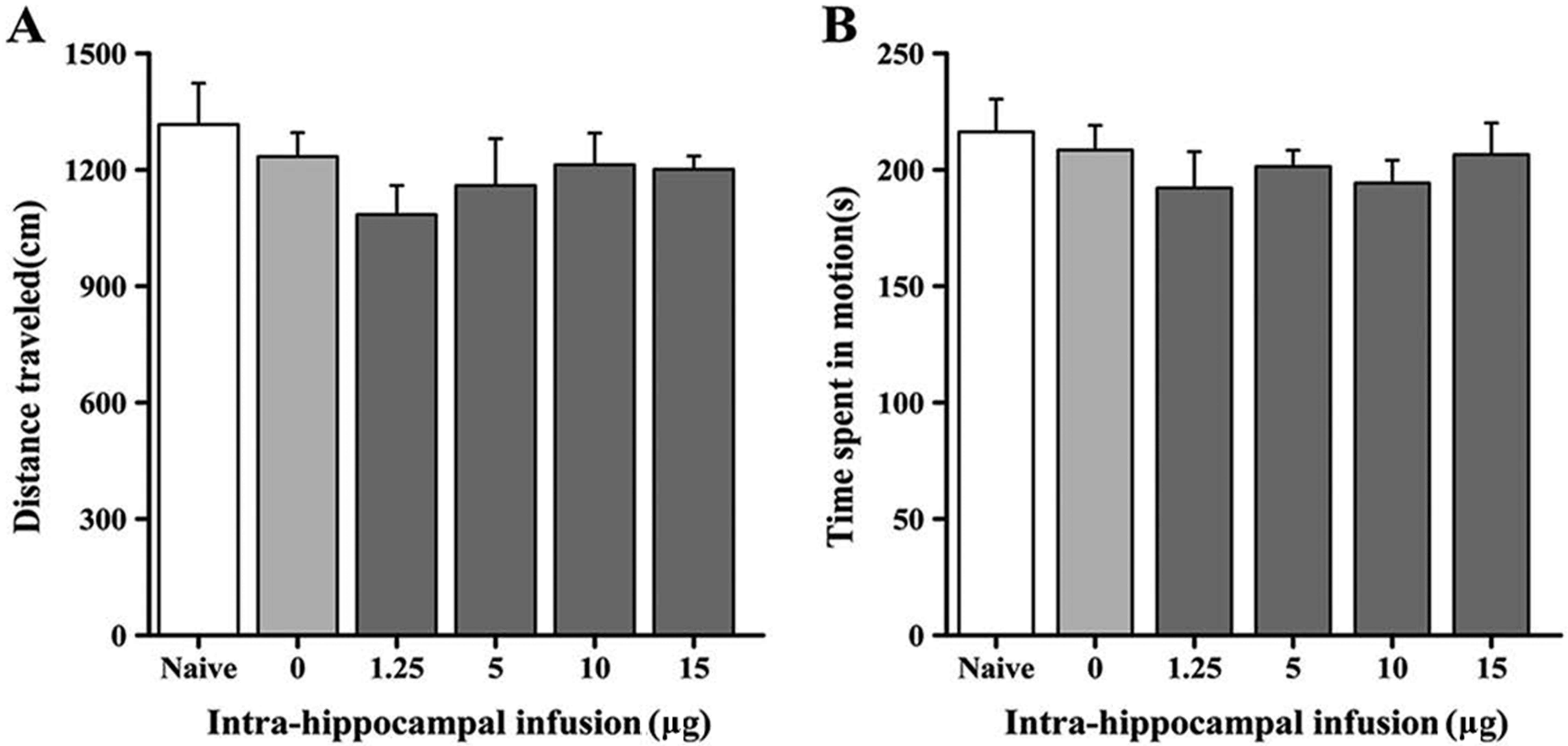


**Fig. S2. Effects of bilateral microinjections of different doses of propranolol into the dorsal hippocampus on the exploratory activity in response to a novel condition.** There were no significant effects of different injections on overall locomotion -- either the distance travelled or time spent in motion (n = 5 per group).
