# Supplementary material for: Propranolol can induce PTSD‐like memory impairments in rats
Source: Brain Behav. 2018 Jan 18;8(2):e00905. doi: 10.1002/brb3.905 (PMC5822589; doi:10.1002/brb3.905)
Supplement: Supplementary file 3 [file BRB3-8-e00905-s003.pdf]

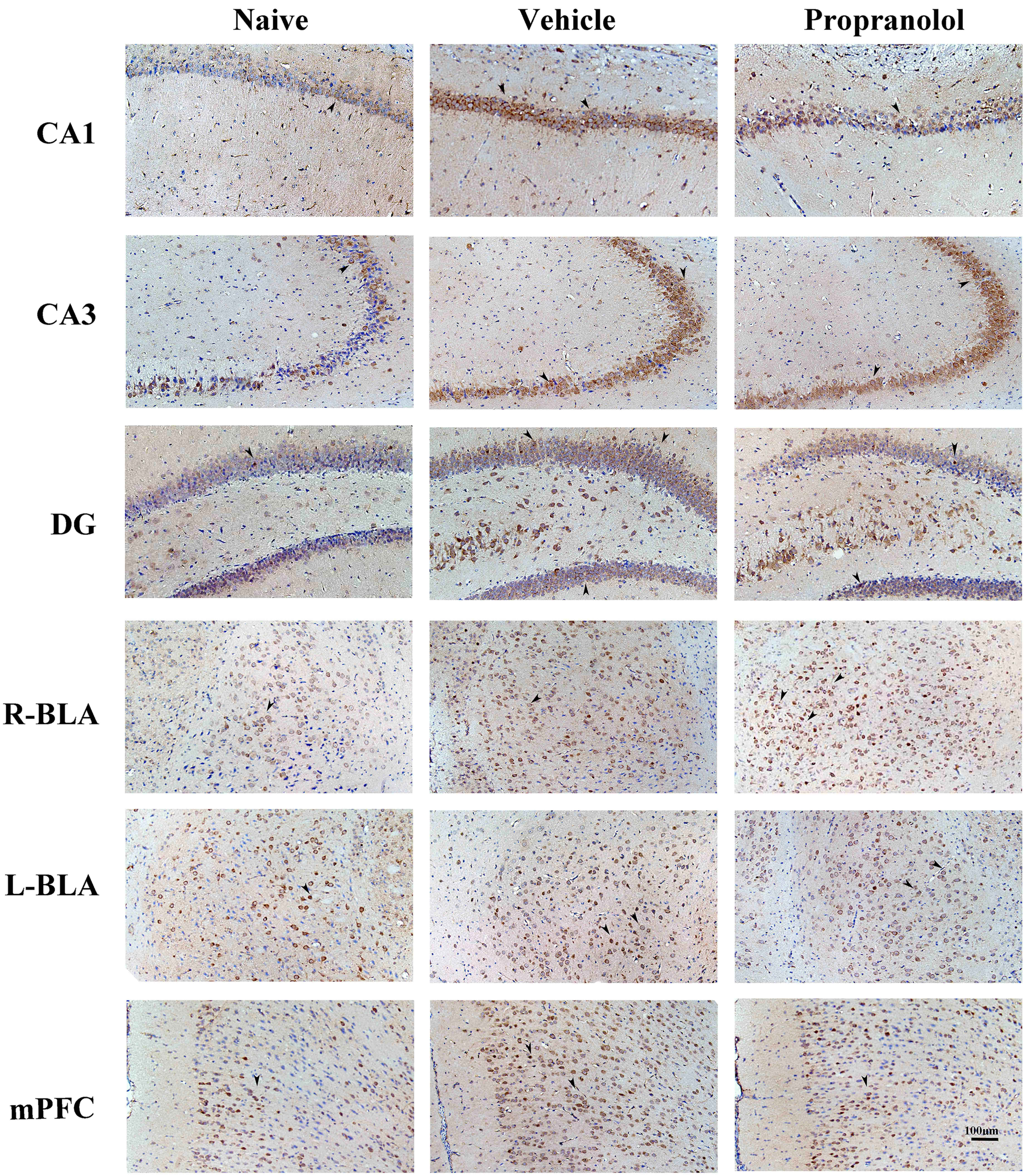

Fig. S3. Representative microphotographs of c-Fos immunoreactivity in specific brain areas of rats that received intra-dorsal hippocampal injections of saline or propranolol (5 μg/0.5 μl) immediately after conditioning with 1.4-mA foot shocks.
